# Impact of "Sambhav" Program (Financial Assistance and Counselor Services) on Hepatitis C Pegylated Interferon Alpha Treatment Initiation in India

**DOI:** 10.15171/ijhpm.2018.84

**Published:** 2018-09-11

**Authors:** Ajit Sood, Vandana Midha, Shivalingappa S. Halli, Vikram Narang, Ramit Mahajan, Varun Mehta, Kirandeep Kaur, Vijay Surlikar, Subodh Kanchi, Dharmatma Singh

**Affiliations:** ^1^Department of Gastroentrology, Dayanand Medical College and Hospital (DMCH), Ludhiana, India.; ^2^Department of Internal Medicine, Dayanand Medical College and Hospital (DMCH), Ludhiana, India.; ^3^University of Manitoba, Winnipeg, MN, Canada.; ^4^Department of Pathology, Dayanand Medical College & Hospital (DMCH), Ludhiana, India.; ^5^Department of Pharmacology, Dayanand Medical College and Hospital, Ludhiana, India.; ^6^MSD Pharmaceutical Pvt Ltd, Bandra, India.

**Keywords:** Sambhav Program, Hepatitis C, Pegylated Interferon Gamma, Treatment Access, India

## Abstract

**Background:** Financial constraints, social taboos and beliefs in alternative medicine are common reasons for delaying or not considering treatment for hepatitis C in India. The present study was planned to analyze the impact of non-banking interest free loan facility in patients affected with hepatitis C virus (HCV) in North India.

**Methods:** This one year observational, retrospective study was conducted in Department of Gastroenterology (January 2012-December 2013), Dayanand Medical College and Hospital Ludhiana, to evaluate the impact of program titled "Sambhav" (which provided non-banking financial assistance and counselor services) on treatment initiation and therapeutic compliance in HCV patients. Data of fully evaluated patients with chronic hepatitis, and/or cirrhosis due to HCV infection who were treated with Peginterferon alfa and ribavirin (RBV) combination during this duration (2012- 2013) was collected from patient medical records and analyzed. In the year 2012, eligible patients who were offered antiviral treatment paid for treatment themselves, while in 2013, ‘Sambhav’ program was launched and this provided interest free financing by non-banking financial company (NBFC) for the treatment of HCV in addition to free counselor services for disease management. The treatment initiation and compliance rates were compared between the patients (n = 585) enrolled in 2013 who were offered ‘Sambhav’ assistance and those enrolled in 2012 (n = 628) when ‘Sambhav’ was not available.

**Results:** Introduction of Sambhav program improved the rates of treatment initiation (59% in 2013 vs. 51% in 2012, P=.004). Of the 585 eligible patients offered ‘Sambhav’ assistance in 2013, 233 patients (39.8%) applied but 106/233 (45.4%) received assistance. Antiviral therapy was started in 93/106 (87.7%) of these patients, while only 52 (42.5%) of 127 patients whose applications were rejected underwent treatment. Compliance to antiviral therapy also improved with the introduction of ‘Sambhav’ program (87.7% vs. 74.1%, P=.001).

**Conclusion:** ‘Sambhav’ program had significant impact on the initiation of antiviral therapy by overcoming the financial hurdles. The free counselor services helped to mitigate social taboos and imparted adequate awareness about the disease to the patients. Initiatives like ‘Sambhav’ can be utilized for improving healthcare services in developing countries, especially for chronic diseases.

## Introduction

Provision of healthcare security or healthcare insurance in developing countries like India continues to be one of the most important unresolved policy issues. A majority of the patients affected with chronic illnesses spend a large percentage of their income on health-related expenditure, especially when they seek in-patient care for major health issues. Further, the high incidence of sickness cuts into their budget in two different ways: they need to spend large amount of money for treatment and are unable to earn money while they are under treatment. It is estimated that at least 24% of all Indians hospitalized fall below the poverty line because of hospitalization and out of pocket spending on hospital care.^1-4^

Hepatitis C virus (HCV) infection is estimated to affect 0.5%–1.5% of Indian population showing much higher prevalence in some areas of northeast India, in some tribal populations and in certain parts of Punjab. The long-term implications of HCV infection are variable, ranging from minimal histological changes to extensive fibrosis and cirrhosis with or without hepatocellular carcinoma.^5^ The primary goal of HCV therapy is to cure the infection. In India approximately 14 million patients are affected with chronic hepatitis C (CHC), but treatment rate is as low as 1% when compared with diagnosis rate of 6%. The lack of disease awareness, its consequences and financial constraints are the major barriers which prevent successful treatment outcomes.^6^

Until 2011, the combination of pegylated interferon (PEG-IFN) and ribavirin (RBV) for 24 or 48 weeks was the standard of care for CHC. With this regimen, sustained virological response (SVR) rates were higher in patients infected with HCV genotypes 2, 3, 5, and 6 (up to about 80%) and intermediate SVR rates (40%–50%) were achieved in those with HCV genotypes 1 and 4. During the study period 2012-2013, the standard of care was peg-interferon with RBV and the average cost of the entire treatment was between 150 000-300 000 INR (US$2345-4695).^5-7^ The newer direct acting antivirals (DAAs) which are highly efficacious and cheaper were introduced in India after the study period, ie, after 2014.^8^ The prevalence of CHC in Punjab and Haryana has been reported to be as high as 4%. Treatment with Peg-IFN alpha and RBV was costly, and only 0.1% of the patients could afford treatment. In addition to financial constraints, fear of side effects, several myths about HCV among the people and faith in alternative medicines limited the acceptance of therapy with anti-virals.

To address these issues, ‘Sambhav’ program was launched in 2013, offering interest free loan for the needy patients for the HCV treatment, monitoring compliance and addressing myths through counselor services. We hereby present our analysis of impact of this program on the initiation of treatment and subsequent compliance with Peg-IFN and RBV in patients with CHC.

## Methods

### Setting

This was an observational, retrospective study conducted between January 2012 and December 2013 in Department of Gastroenterology, Dayanand Medical College and Hospital (DMC & H), Ludhiana, Punjab, India. The aim of the study was to evaluate impact of financial support on treatment access and compliance in CHC. During this period the available treatment for patients with chronic hepatitis or cirrhosis due to HCV included PEG-IFN alpha in combination with RBV. The acceptance and compliance to antiviral treatment in the patients treated for CHC in 2012 (with self-payment) and 2013 (with support from ‘Sambhav’ program) was retrospectively analyzed.

### The “Sambhav” Program

The ‘Sambhav’ (Hindi word, meaning it is possible) program, launched in 2013, was a novel collaboration between a hospital (Dayanand Medical College and Hospital Ludhiana), Merck pharmaceutical company (MSD) and a financing institution (Fullereton, India) to address the needs of the patients with CHC. This program had two components: first; financing by way of loan and second; provision of free counseling services for proper disease management.

The main objective of Sambhav program was to overcome cash flow issues, remove any excess financial burden on patients choosing this program and remove patient liability if there was no cure. Under the financing program, eligible patients got an interest free, unsecured financing from a non-banking financial company (NBFC), for the treatment of hepatitis C. The loan provided under the financing scheme was payable over period of 2-4 years (depending upon the affordability and preference of the patients) and the payment was to be done in the form of equated monthly installments (EMIs), (calculated using Fullerton software, then rebate given toward end of loan). The loans were provided to traditionally difficult to fund profiles eg, farmers and shop owners etc. Other incentives included zero extra charge and easier loan terms, simplified documentation requirements and waiver of the outstanding EMIs in case of non-cure (defined as drug non-responders at 12 weeks and patients not achieving end of treatment response [ETR] or SVR after completion of therapy).

For disease management the main objectives of HCV counsellors were to optimize the chances of achieving cure of CHC by removing myths, if any, and to help patients understand the importance of treatment and completing the full course. Free counselor services, ‘Saarthi’ (Sanskrit word for charioteer, signifying the one who helps one to pass through an obstacle) were started to implement this. These included in-person and phone counseling services, free diagnostic tests and assistance in procurement of the loan (Figure).

**Figure:**
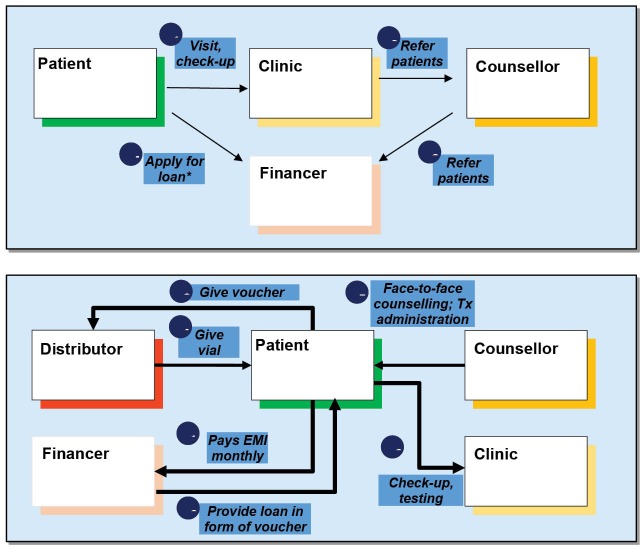
Depicting the Flow of Patient Enrolment, loan Sanction and Follow up in Sambhav Program.

### Patients and Treatment

All patients who had been found to be HCV positive had been assessed for the status of liver disease (liver biochemical tests, ultrasonography and biopsy, if indicated) and viral characteristics (HCV RNA levels and genotype). Those found to be fit for therapy (patients with advanced liver disease, depression, cardiopulmonary disease, renal failure were excluded) had been offered PEG-IFN and RBV. Those who started anti-viral therapy had been monitored for compliance, efficacy and adverse events. Compliance of treatment was noted as per patients statements. The patients were followed up every 4 weeks in clinic of the gastroenterologist and were weekly monitored for compliance and treatment related queries by the counselor either personally or telephonically. In the year 2012, the patients accepting treatment had to pay for the treatment themselves, while financial assistance and free counseling was provided by the Sambhav program in 2013.

In 2013, the confirmed cases for HCV requiring treatment with PEG-IFN and RBV were introduced to the ‘Sambhav’ program by the counselor/treating physician. Patients offered ‘Sambhav’ program included (*a*) All confirmed patients (age <60 years) for HCV requiring treatment (*b*) monthly documented income >INR 10 000 (US$157). The other prerequisites for financial assistance included (*i*) one loan per family; (ii) minimum amount of loan INR 100 000 (US$1565); (*iii*) payment of an interest if loan repayment tenure was more than 2-4 years. The exclusion criteria were (*a*) age >60 years (*b*) single patients (widow/widower, divorcee), and (*c*) monthly documented income <INR 10 000 (US$157).

For analysis the patients were divided into 2 groups:

- Group A: CHC patients presenting to the DMC & H after launch of ‘Sambhav’ (in year 2013).
- Group A1: Patients who received the ‘Sambhav’ assistance.
- Group A2: Patients who did not receive the ‘Sambhav’ assistance either because they did not apply or because their application was rejected.
- Group B: Confirmed CHC patients presenting to the clinics prior to SAMBHAV (in year 2012). A designated CRO (Central Record Officer) captured, checked, stored and analyzed the data. CRO followed internal SOPs (Standard operating procedures). The data was transferred to the principal investigator after termination of the study. Quality control audits of all key safety and effectiveness data in the database were made prior to locking the database.

### Data Analysis

Data analysis was done using SPSS and was summarized using descriptive summary statistics (mean and standard deviation). Bi-variate analyses were performed to test the relationship between the background characteristics of the patients and the outcome variables such as the HCV treatment initiation and compliance using chi-square tests. *P* values <.05 were considered to indicate statistical significance. The multivariate logistic regression was used in order to control for the important background factors to verify the bi-variate relationships between the interest free loan through ‘Sambhav’ and the outcome variables such as the HCV treatment initiation and compliance.

## Results

In 2012 a total of 628 patients (self-payment, no ‘Sambhav’ program) and in 2013, 585 patients (under ‘Sambhav’ program) were included. The baseline characteristics of the patients are listed in Table 1.

**Table 1:** Baseline Patient Characteristics

**Variable(s)**	**Group = 2012** ** (n = 628)**	**Group = 2013** ** (n = 585)**
Age (y) (mean ± SD)	43.1 ± 13.2	42.9 ± 11.7
Height (cm) (mean ± SD)	168.6 ± 8.2	168.4 ± 8.4
Weight (kg) (mean ± SD)	70.1 ± 14.2	70.1 ± 13.4
Gender, No. (%)		
Male	422 (67.2)	391 (66.8)
Female	206 (32.8)	194 (33.2)
Education, No. (%)		
Elementary education	241 (38.4)	192 (32.8)
Secondary	304 (48.4)	307 (52.5)
Graduate and above	83 (13.2)	86 (14.72)
Monthly Income, No. (%)		
<10K	255 (40.6)	135 (23.1)
10-25K	255 (40.6)	293 (50.1)
>25K	112 (17.8)	151 (25.8)
Missing data	06 (1.0)	06 (1.0)
Occupation, No. (%)		
Unemployed	230 (36.6)	189 (32.3)
Farmer and shop owner	331 (52.7)	335 (57.3)
White-collar jobs	63 (10.0)	59 (10.1)
Missing data	04 (0.6)	02 (0.3)
Alcohol consumption, No. (%)	180 (28.7)	174 (29.7)
Drug abuse, No. (%)	43 (6.8)	45 (7.7)
Smokers, No. (%)	09 (1.4)	36 (6.2)

Treatment for CHC was initiated in 318/628 patients (51%) in 2012. All these patients had to make the payment for anti-viral therapy from their own pocket. After the launch of ‘Sambhav’ program in 2013, the rate of treatment initiation significantly increased to 59% (344/585, *P* = .004), however there was no significant change in the duration of treatment or rates of compliance to therapy (Table 2).

**Table 2:** Treatment Initiation Duration and Compliance

**Variable (s)**	**Group = 2012 (N=628**	**Group = 2013 (N=585)**	***P*** ** Value**
Patients initiating treatment, No. (%)	318 (50.6)	344 (58.8)	<.001^a^
Treatment duration (wk), (mean ± SD)	25.9 ± 10.5	26.7 ± 10.6	>.5^b^
Treatment compliance (%)	233/318 (73.3)	271/344 (78.8)	>.05

^a^Based on chi-square test. ^b^Based on independent *t* test.

Of the 233/585 (39.8%) of the patients who applied for the ‘Sambhav’ program, 106 (45.4%) received the financial assistance. The applications of remaining 127 patients were rejected due to various reasons (age >60 years [05/127, 3.9%], low income [52/127, 40.9%], non-compliance after the initiation of loan process [70/127, 55.1%]).

Among the patients who applied for the ‘Sambhav’ program, the rate of treatment initiation was higher (93/106, 87.7%) in patients who got financial assistance as compared to the patients who paid for the treatment by themselves due to failure to enroll in the ‘Sambhav’ program (52/127, 42.5%, *P* <.001).

In 2013, a total of 344 patients were treated with Peg-INF aipha and RBV. Of the 344 patients only 145/344 (42.5%) had applied for Sambhav program while 57.5% (199/344) did not apply for this novel initiative. However more patients (145/233, 62.2% vs. 199/353, 56.5%) initiated treatment when exposed to Sambhav initiative (Table 3). Thus, the introduction of ‘Sambhav’ program increased the likelihood of treatment initiated by an odds ratio of 10 (Tables 4 and 5).

**Table 3:** Treatment Initiation as Compliance in Patients Applying for Financial Assistance (Group = 2013)

	**Financial Assistance Granted (n = 106)**	**Financial Assistance Not-granted (n = 127)**	***P*** ** Value**
Started treatment, (n = 233) No. (%)	93 (87.7)	54 (42.5)	<.001
Treatment compliance, (n = 147) No. (%)	81 (87.1)	40 (74.1)	<.05

**Table 4:** Logistic Regression (Patient Initiating Treatment as Dependent Variable)

	**B**	**SE**	**Wald**	***df***	***P*** ** Value**	**Exp (B)**
Age	0.000	0.016	0.000	1	1.000	1.000
Gender	0.227	0.605	0.140	1	.708	1.254
Residential status (0=U/1=R)	-0.806	0.462	3.051	1	.081	0.447
Education score (0 = elementary education)	‏-	‏-	1.843	2	.398	‏-
Education score (1 = secondary)	1.068	0.799	1.788	1	.181	2.910
Education score (2 = ≥ graduate)	0.671	0.681	0.970	1	.325	1.956
Monthly income (0 = <10K)	‏-	‏-	13.259	2	.001	‏-
Monthly income (1 = 10-25K)	-2.337	0.647	13.048	1	.000	0.097
Monthly income (2 = >25K)	-1.674	0.562	8.871	1	.003	0.188
Occupation score (0 = Unemployed)	‏-	‏-	1.295	2	.523	‏-
Occupation score (1 = shopown and farmers)	0.872	0.969	0.811	1	.368	2.392
Occupation score (2 = white-collar jobs)	0.207	0.838	0.061	1	.804	1.231
Family history (0=N/1=Y)	0.434	0.414	1.100	1	.294	1.544
Loan application accepted (0=N/1=Y)	2.350	0.412	32.522	1	.000	10.489
Constant	0.498	1.010	0.243	1	.622	1.646

Abbreviation: SE, standard error.

**Table 5:** Variables in the Equation With Respect to Table 4

		**B**	**SE**	**Wald**	***df***	***P *** **Value**	**Exp(B)**	**95% CI for EXP(B)**	
								**Lower**	**Upper**
Step 1	Age	0.000	0.016	0.000	1	1.000	1.000	0.968	1.033
	Gender (1)	-0.227	0.605	0.140	1	.708	0.797	0.243	2.611
	Residential status (1)	0.806	0.462	3.051	1	.081	2.239	0.906	5.534
	Education score	-	-	1.843	2	.398	-	-	-
	Education score (1)	1.068	0.799	1.788	1	.181	2.910	0.608	13.927
	Education score (2)	0.671	0.681	0.970	1	.325	1.956	0.515	7.435
	Monthly income	‏-	‏-	13.259	2	.001	‏-	‏-	‏-
	Monthly income (1)	-2.337	0.647	13.048	1	.000	0.097	0.027	0.343
	Monthly income (2)	-1.674	0.562	8.871	1	.003	0.188	0.062	0.564
	Occupation score	-	-	1.295	2	.523	-	-	-
	Occupation score (1)	0.872	0.969	0.811	1	.368	2.392	0.358	15.968
	Occupation score (2)	0.207	0.838	0.061	1	.804	1.231	0.238	6.357
	Family history (1)	-0.434	0.414	1.100	1	.294	0.648	0.288	1.458
	Accepted YN (1)	-2.350	0.412	32.522	1	.000	0.095	0.043	0.214
	Constant	2.703	0.949	8.107	1	.004	14.927	-	-

Abbreviations: SE, standard error; YN, yes/no.

The loan was waived of in 12/93 (12.9%) patients loan as they did not respond to drugs at 12 weeks (failed rapid virological response, RVR). The treatment compliance rate also improved with the financial help and counseling from the ‘Sambhav’ program (87.1% with ‘Sambhav’ vs. 74.1% without ‘Sambhav’). On bivariate analysis between 2012 and 2013 the odds ratio for the SAMBHAV interest free loan after controlling for the patients’ socio-economic status indicates likelihood of increase in the compliance by nearly 4 times (Tables 6 and 7).

**Table 6:** Logistic Regression (Compliance to Treatment as Dependent Variable)

	**B**	**SE**	**Wald**	***df***	***P*** ** Value**	**Exp (B)**
Age	-0.060	0.024	6.223	1	.13	0.941
Gender	1.562	0.895	3.046	1	.081	4.767
Residential status (0=U/1=R)	0.746	0.567	1.733	1	.188	2.108
Education score (0 = elementary education)	‏-	‏-	2.941	2	.230	‏-
Education score (1 = secondary)	0.316	1.112	0.081	1	.776	1.371
Education score (2 = ≥ graduate)	-0.713	0.950	0.565	1	.452	0.490
Monthly income (0 = <10K)	‏-	‏-	0.743	2	.690	‏-
Monthly income (1 = 10-25K)	0.020	0.915	0.001	1	.982	1.021
Monthly income (2 = >25K)	-0.465	0.612	0.578	1	.447	0.628
Occupation score (0 = Unemployed)	‏-	‏-	3.965	2	.138	‏-
Occupation score (1 = shopown and farmers)	1.990	1.202	2.742	1	.098	7.312
Occupation score (2 = white-collar jobs)	0.258	1.000	0.067	1	.796	1.294
Family history (0=N/1=Y)	-0.287	0.591	0.236	1	.627	0.751
Loan application accepted (0=N/1=Y)	1.262	0.542	5.417	1	.020	3.532
Constant	1.980	1.371	2.086	1	.149	7.242

Abbreviations: SE, standard error; YN, yes/no.

**Table 7:** Variables in the Equation With Respect to Table 6

		**B**	**SE**	**Wald**	***df***	***P *** **Value**	**Exp(B)**	**95% CI for EXP(B)**	
								**Lower**	**Upper**
Step 1	Age	-0.060	0.024	6.223	1	.013	0.941	0.898	0.987
Gender (1)	-1.562	0.895	3.046	1	.081	0.210	0.036	1.212
Residential status (1)	-0.746	0.567	1.733	1	.188	0.474	0.156	1.440
Education score	-	-	2.941	2	.230	-	-	-
Education score (1)	0.316	1.112	0.081	1	.776	1.371	0.155	12.127
Education score (2)	-0.714	0.950	0.565	1	.452	0.490	0.076	3.150
Monthly income	‏-	‏-	0.743	2	.690	‏-	‏-	‏-
Monthly income (1)	0.020	0.915	0.001	1	.982	1.021	0.170	6.131
Monthly income (2)	-0.465	0.612	0.578	1	.447	0.628	0.189	2.084
Occupation score	-	-	3.965	2	.138	-	-	-
Occupation score (1)	1.990	1.202	2.742	1	.098	7.312	0.694	77.063
Occupation score (2)	0.258	1.000	0.067	1	.796	1.294	0.182	9.197
Family history (1)	0.287	0.591	0.236	1	.627	1.332	0.418	4.243
Accepted YN (1)	-1.262	0.542	5.417	1	.020	0.283	0.098	0.819
Constant	5.263	1.480	12.641	1	.000	192.966	-	-

Abbreviations: SE, standard error; YN, yes/no.

## Discussion

In 2013, 585 patients were enrolled and offered ‘Sambhav’ assistance and the treatment initiation and compliance rates were compared with 2012 group (n = 628) when ‘Sambhav’ was not available. Of the 233 patients (39.8%) who applied for ‘Sambhav’ program, 45.4% (106/233) received the assistance while 127 applications were rejected due to various reasons. There was a significant difference between the two groups in HCV treatment initiation (51% in 2012 and 59% in 2013) (*P* = .004). A majority 87.7% (93/106) of the patients who received ‘Sambhav’ assistance initiated the treatment compared to only 42.5% (52/127) among those who were offered ‘Sambhav’ support but did not get it due to various reasons. The compliance rates were also better (87.7% vs. 74.1%) when compared groups with and without ‘Sambhav’ support.

HCV related liver diseases are major healthcare burden in Punjab. There are many patient, provider, government and payer factors which effectively prevent delivery of HCV care. Patient-related factors are a common source of treatment deferral and include limited awareness, poor compliance to physician recommendations, economic or social pressures, treatment fears, psychiatric disease and injection drug use. Specific factors identified as barriers to medication compliance among patients with low socioeconomic status were high medication costs and poor understanding of medication instructions. Also, the patients lack knowledge about the urgency of the initiating therapy, adverse events and existence of cure for this disease with successful treatment. There is stigma and many myths associated with HCV, leading patients seeking alternative remedies through quacks. Therefore, these patients need counseling about various aspects of disease and therapy.^5-7^

In India, a majority of the financial assistance is provided by the banks but recently NBFC’s are becoming popular among the general public and loan seekers. The banks in India are incorporated by the Banking Companies Act, whereas NBFCs are incorporated by the Companies Act of 1956. NBFCs can just make investment or lend, they do not accept demand deposits. But when it comes to borrowing loan most prefer NBFCs over banks and the reason for this is banks have hard rules and require more time to approve or sanction a loan. On the other hand, NBFCs ensure quicker processing and necessary loan amount is disbursed within days. Though rate of interest is high at NBFCs most of the times as compared to banks, borrowers still prefer to take loans from NBFC considering the ease of getting loan and lesser complications. Moreover, individuals with poor credit rating are considered to be high-risk and generally do not get loans from banks. Unless the credit score is above 600-650, it is very difficult to get a loan sanctioned from banks. NBFCs on the other hand offer loans to individuals with low credit score and have brought down the interest rates to either equal to bank lending rates or at times even lower than that.^3,4,7^ Thus, with all the other benefits when rate of interest is also lowered, and borrowers find this easier and affordable. This has also resulted in lower EMI for the borrowers.

In 2012, Merck & Co. launched its pilot Hepatitis Financing Mechanism, or ‘Sambhav’ Program in the state of Punjab in India. Market research revealed that many patients could not afford the cost of treatment for hepatitis C. So in response, for patients with limited or no insurance coverage, the company developed an innovative financing model for its hepatitis C treatment in high prevalence areas for HCV infection in North India. This novel program had two components: free counselor services for disease management and financing by way of loan. Under the financing program, eligible patients get interest free, unsecured financing from NBFC, for the treatment of hepatitis C and counselor services were rendered to address various concerns and myths of patients regarding therapy and disease. This was a unique collaboration between a hospital/clinic, pharmaceutical company and financing institution to develop a new business model to address a major unmet patient need – access to medicines by reducing cash flow burden. The benefits of providing financial support were observed as more number of patients initiated treatment.

The ultimate aim of any prescribed medical therapy is to achieve certain desired outcomes in the patients concerned. These desired outcomes are part and parcel of the objectives in the management of the diseases or conditions. However, despite all the best intention and efforts on the part of the healthcare professionals, those outcomes might not be achievable if the patients are non-compliant.^9-11^ This shortfall may also have serious and detrimental effects from the perspective of disease management. Hepatitis C infection is a chronic liver disease requiring treatment for longer duration and regular follow ups with their treating physicians. Moreover, continuous motivation is required to start treatment considering the social stigma the HCV carries along with it especially in North India. The ‘Sambhav’ program provided counselor services through its project Saarthi which lead to statistically significant (*P *= .001) difference in the treatment compliance between those who received ‘Sambhav’ assistance and those who did not (87% among those who received compared to 74% those who did not). It may be recalled that the impact of ‘Sambhav’ program on the compliance was not significant when we did not differentiate between those actually received the financial assistance and those who did not. This fact highlights the importance of counselor services which allow patients to understand the disease better, treatment related myths are attended; patients are kept motivated to continue treatment, and are also made aware of any therapy related side effects.

## Conclusion

HCV is prevalent in Northern India especially in the state of Punjab. Financial constraints and lack of knowledge are the barriers for not seeking treatment leading to serious liver diseases. As a response to this, an innovative program was initiated in high prevalence states including Punjab not only to provide unsecured interest free loan and also disease management. The treatment initiation rate of the HCV increased and treatment compliance also improved significantly when provided the ‘Sambhav’ support. In this context, the ‘Sambhav’ program experience has important implications for policy development.

## Acknowledgements

The authors will also like to acknowledge the content and editorial support provided Mr. Sandeep K. Bhat at medONE Pharma Solution, New Delhi, India, during preparation of this manuscript.

## Ethical issues

The ethical clearance from the institutional board was sought and the study was conducted in accordance with the ICH Harmonized Tripartite Guidelines for Good Clinical Practice (GCP), with applicable local regulations; Guidelines for Good Pharmacoepidemiology Practices (GPP) of the International Society for Pharmacoepidemiology, and with the ethical principles laid down in the Declaration of Helsinki.

## Competing interests

Authors declare that they have no competing interests.

## Authors’ contributions

Concept and design: AS, VanM, and VarM. Aquisition of data: DS, KK, and VS. Analysis and interpretation of data: AS, VanM, SSH, and VN. Drafting of manuscript: AS, VN, and RM. Critical revision of manuscript for intellectual content: AS, VanM. Statistical aanalysis: SSH and VS. Obtaining funding: AS, SSH, and VS. Administrative, material, and technical support: SK and DS.

## Authors’ affiliations

^1^Department of Gastroentrology, Dayanand Medical College and Hospital (DMCH), Ludhiana, India. ^2^Department of Internal Medicine, Dayanand Medical College and Hospital (DMCH), Ludhiana, India. ^3^University of Manitoba, Winnipeg, MN, Canada. ^4^Department of Pathology, Dayanand Medical College & Hospital (DMCH), Ludhiana, India. ^5^Department of Pharmacology, Dayanand Medical College and Hospital, Ludhiana, India. ^6^MSD Pharmaceutical Pvt Ltd, Bandra, India.

## Key messages

**Implications for policy makers**

- Heavy financial burden required for treatment of chronic diseases is a major deterrent for seeking medical aid.
- Lack of disease awareness also contributes towards delayed treatment in developing countries.

**Implications for public**

Hepatitis C infection is the most common cause of chronic liver disease. The severity of hepatitis C, its progression, and response to therapy may vary depending on the genotype.

The common modes of transmission of hepatitis C infection are blood transfusion, IV drug use, unsafe therapeutic injection, and health care-related procedures. In India blood transfusion and unsafe injection practices are predominant modes of hepatitis C transmission.
